# Comparative Performance of Private and Public Healthcare Systems in Low- and Middle-Income Countries: A Systematic Review

**DOI:** 10.1371/journal.pmed.1001244

**Published:** 2012-06-19

**Authors:** Sanjay Basu, Jason Andrews, Sandeep Kishore, Rajesh Panjabi, David Stuckler

**Affiliations:** 1 Department of Medicine, University of California, San Francisco, California, United States of America; 2 Division of General Internal Medicine, San Francisco General Hospital, San Francisco, California, United States of America; 3 Department of Public Health and Policy, London School of Hygiene & Tropical Medicine, London, United Kingdom; 4 Division of Infectious Diseases, Massachusetts General Hospital, Boston, Massachusetts, United States of America; 5 Tri-Institutional MD-PhD Program, Weill Cornell Medical College/Rockefeller University/Sloan-Kettering Institute, New York, New York, United States of America; 6 Division of Global Health Equity, Brigham and Women's Hospital, Harvard Medical School, Boston, Massachusetts, United States of America; 7 Department of Sociology, Cambridge University, Cambridge, United Kingdom; King's College London, United Kingdom

## Abstract

**Introduction:**

Private sector healthcare delivery in low- and middle-income countries is sometimes argued to be more efficient, accountable, and sustainable than public sector delivery. Conversely, the public sector is often regarded as providing more equitable and evidence-based care. We performed a systematic review of research studies investigating the performance of private and public sector delivery in low- and middle-income countries.

**Methods and Findings:**

Peer-reviewed studies including case studies, meta-analyses, reviews, and case-control analyses, as well as reports published by non-governmental organizations and international agencies, were systematically collected through large database searches, filtered through methodological inclusion criteria, and organized into six World Health Organization health system themes: accessibility and responsiveness; quality; outcomes; accountability, transparency, and regulation; fairness and equity; and efficiency. Of 1,178 potentially relevant unique citations, data were obtained from 102 articles describing studies conducted in low- and middle-income countries. Comparative cohort and cross-sectional studies suggested that providers in the private sector more frequently violated medical standards of practice and had poorer patient outcomes, but had greater reported timeliness and hospitality to patients. Reported efficiency tended to be lower in the private than in the public sector, resulting in part from perverse incentives for unnecessary testing and treatment. Public sector services experienced more limited availability of equipment, medications, and trained healthcare workers. When the definition of “private sector” included unlicensed and uncertified providers such as drug shop owners, most patients appeared to access care in the private sector; however, when unlicensed healthcare providers were excluded from the analysis, the majority of people accessed public sector care. “Competitive dynamics” for funding appeared between the two sectors, such that public funds and personnel were redirected to private sector development, followed by reductions in public sector service budgets and staff.

**Conclusions:**

Studies evaluated in this systematic review do not support the claim that the private sector is usually more efficient, accountable, or medically effective than the public sector; however, the public sector appears frequently to lack timeliness and hospitality towards patients.

*Please see later in the article for the Editors' Summary*

## Abstract 2

A systematic review conducted by Sanjay Basu and colleagues reevaluates the evidence relating to comparative performance of public versus private sector healthcare delivery in low- and middle-income countries.

## Editors' Summary

**Background:**

Health care can be provided through public and private providers. Public health care is usually provided by the government through national healthcare systems. Private health care can be provided through “for profit” hospitals and self-employed practitioners, and “not for profit” non-government providers, including faith-based organizations.

There is considerable ideological debate around whether low- and middle-income countries should strengthen public versus private healthcare services, but in reality, most low- and middle-income countries use both types of healthcare provision. Recently, as the global economic recession has put major constraints on government budgets—the major funding source for healthcare expenditures in most countries—disputes between the proponents of private and public systems have escalated, further fuelled by the recommendation of International Monetary Fund (an international finance institution) that countries increase the scope of private sector provision in health care as part of loan conditions to reduce government debt. However, critics of the private health sector believe that public healthcare provision is of most benefit to poor people and is the only way to achieve universal and equitable access to health care.

**Why Was This Study Done?:**

Both sides of the public versus private healthcare debate draw on selected case reports to defend their viewpoints, but there is a widely held view that the private health system is more efficient than the public health system. Therefore, in order to inform policy, there is an urgent need for robust evidence to evaluate the quality and effectiveness of the health care provided through both systems. In this study, the authors reviewed all of the evidence in a systematic way to evaluate available data on public and private sector performance.

**What Did the Researchers Do and Find?:**

The researchers used eight databases and a comprehensive key word search to identify and review appropriate published data and studies of private and public sector performance in low- and middle-income countries. They assessed selected studies against the World Health Organization's six essential themes of health systems—accessibility and responsiveness; quality; outcomes; accountability, transparency, and regulation; fairness and equity; and efficiency—and conducted a narrative review of each theme.

Out of the 102 relevant studies included in their comparative analysis, 59 studies were research studies and 13 involved meta-analysis, with the rest involving case reports or reviews. The researchers found that study findings varied considerably across countries studied (one-third of studies were conducted in Africa and a third in Southeast Asia) and by the methods used.

Financial barriers to care (such as user fees) were reported for both public and private systems. Although studies report that patients in the private sector experience better timeliness and hospitality, studies suggest that providers in the private sector more frequently violate accepted medical standards and have lower reported efficiency.

**What Do These Findings Mean?:**

This systematic review did not support previous views that private sector delivery of health care in low- and middle-income settings is more efficient, accountable, or effective than public sector delivery. Each system has its strengths and weaknesses, but importantly, in both sectors, there were financial barriers to care, and each had poor accountability and transparency. This systematic review highlights a limited and poor-quality evidence base regarding the comparative performance of the two systems.

**Additional Information:**

Please access these websites via the online version of this summary at http://dx.doi.org/10.1371/journal.pmed.1001244.

- A previous PLoS Medicine study examined the outpatient care provided by the public and private sector in low-income countries
- The WHO website provides more information on healthcare systems
- The World Bank website provides information on health system financing
- Oxfam provides an argument against increased private health care in poor countries

## Introduction

**Figure 1:**
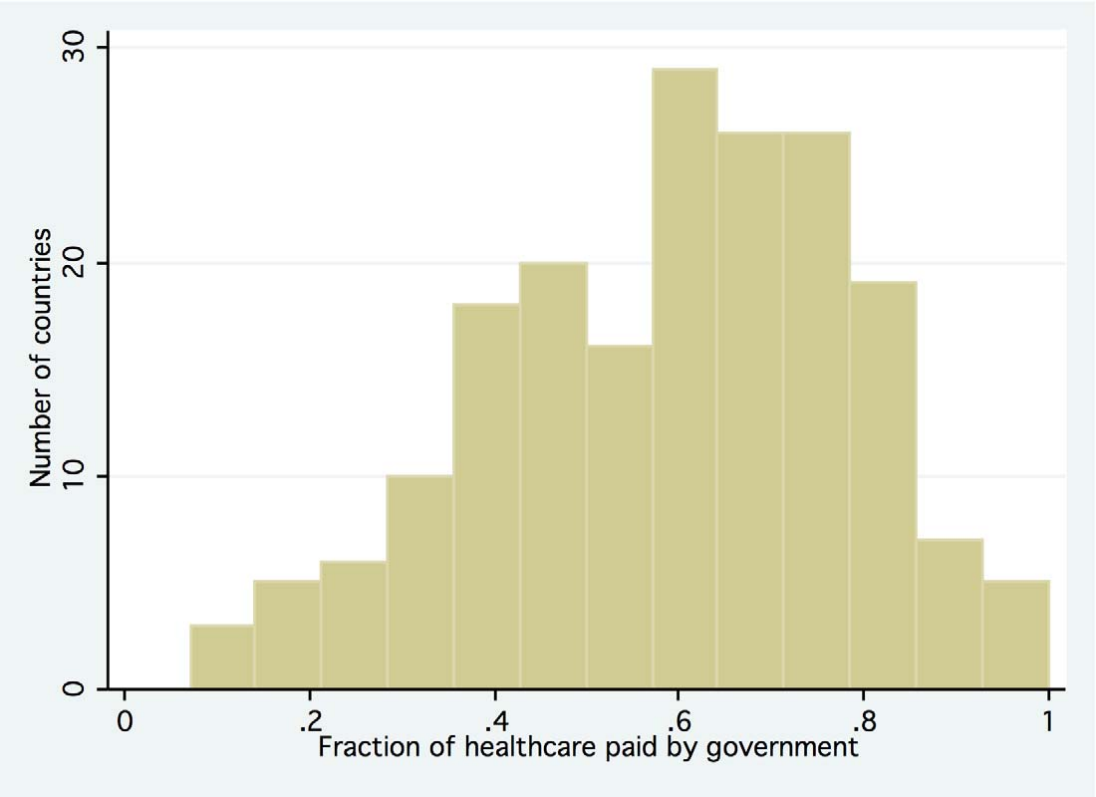
General government expenditure on health as percent of total expenditure on health, 2008. *n* = 190 countries for which data are available. Source: [114].

One longstanding and polarized debate in global health concerns the appropriate role and balance of the public and private sector in providing healthcare services to populations in low- and middle-income countries [1]. In recent years, disputes between the proponents of private and public systems have become particularly heated, as the global economic recession that began in 2007 has placed major constraints on government budgets—the major funding source for healthcare expenditures in most countries (Figure 1) [2]. The International Monetary Fund has recommended that countries increase the scope of private sector provision in health care as part of loan conditions [3], often to reduce government debt [4]. Criticizing such efforts, the international nonprofit organization Oxfam, in its report “Blind Optimism,” concluded that “to achieve universal and equitable access to health care, the public sector must be made to work as the majority provider” [5]. The World Bank responded that it seeks “more pragmatic approaches that build on what is available” by engaging with the private sector in countries where public sector services perform poorly [6]; the Center for Global Development similarly argued that the Oxfam report “ignored the informal sector,” and that poor people “want to go” to private providers and will “persist in doing so” [7].

Generally, this debate has been divided between those seeking universal state-based healthcare availability and those advocating for the private sector to provide care in areas where the public sector has typically failed. Private sector advocates have pointed to evidence that the “private sector is the main provider,” as many impoverished patients prefer to seek care at private clinics [1]. They have suggested that the private sector may be more efficient and responsive to patient needs because of market competition, which they indicate should overcome government inefficiency and corruption [8]. In contrast, public sector advocates have highlighted inequities in access to health care resulting from the inability of the poor to pay for private services. They have noted that private markets often fail to deliver public health goods including preventative services (a “market failure”), and lack coordinated planning with public health systems, required to curb epidemics.

Both sides claim their critics are “ideologically biased” [9],[10] and selectively draw on case reports to defend their viewpoints [5],[7]. However, significant conflicts of interest may apply to both groups [11], as large private international contractors, insurance firms, and non-governmental organizations may benefit from expanding the role of the private sector, while academics who rely on state-funded grant proposals may gain resources from a greater public sector role.

Crucially needed to inform this debate is a systematic review of existing evidence. As Hanson and colleagues note, “A strengthened evidence base on the performance of the public and private health sectors is essential to guide decision-makers towards policy choices that are appropriate for their contexts” [11]. However, in practice, studies comparing the performance of private and public sectors are difficult to implement, for several reasons. First, healthcare services are not universally dichotomized between public and private providers, as some practitioners participate in both state-based and privately owned healthcare delivery systems, and many systems are dually funded or informal. A wide range of arrangements exist for how such expenditures are spent in public versus private clinics, hospitals, and informal settings (see Box 1 for definitions). One example of this complication is the role of informal payments in public facilities. These private–public interactions confound a simplistic comparison between private and public systems. Second, state-based healthcare services and private services have coexisted in many low- and middle-income countries for decades; most countries have a large fraction (but not all) of healthcare expenditures paid for by the state, with most of the remainder paid for by households [12]. In this context, simply defining what is private or public is not straightforward. Private providers are heterogeneous, consisting of formal for-profit entities such as independent hospitals, informal entities that may include unlicensed providers, and nonprofit and non-governmental organizations.

**Box 1. Different Public and Private Healthcare Delivery Agents in Low- and Middle-Income Countries**

**Multinational and national for-profit corporations:** for-profit group practices, sometimes associated with hospitals.

**Formal individual private providers:** individual physicians or other healthcare providers operating in smaller scale healthcare facilities or private pharmacies.

**Informal for-profit providers:** unlicensed, unregulated providers including shop owners, “injectors,” traditional healers, and birth attendants.

**Not-for-profit providers:** civil society, non-governmental, and faith-based groups, charities; and community and social enterprises, with varying degrees of regulation and oversight.

**Public hospitals, health centers, and clinics:** county- and district-level hospitals and clinics, with varying degrees of accessibility and user fees for patients, often having providers that also participate in private sector healthcare delivery.

**Public–private partnerships:** International or national associations that have varying degrees of for-profit or nonprofit status, or collaborations between for-profit and government/nonprofit entities to deliver services. Also have varying user fees for patients and varying levels of public subsidization for delivering healthcare services.

Although these debates have been highly visible, there is a dearth of reviews on the topic. An initial search of prior systematic reviews and meta-analyses in the PubMed database revealed one recent review, evaluating 80 field-based studies that directly and simultaneously compared service quality in ambulatory public and private care clinics [1]. The analysis found that private outpatient clinics often had better drug supplies and responsiveness than public clinics, but the analysis did not assess other dimensions of health system performance (such as accessibility). The review excluded studies of hospitals, case reports, intervention studies (such as how a sector responded to quality improvement programs), or statistical studies of population-level data.

The aim of the current study is to evaluate available data on public and private sector performance across the key domains of health systems competencies. Our goal is to understand how the private or public nature of a given healthcare delivery institution may impact core healthcare delivery goals. We systematically review published data and studies of private and public sector performance in low- and middle-income countries against six health systems themes used by World Health Organization (WHO), adapted from the 2000 World Health Report [13]. The six themes are as follows: accessibility and responsiveness; quality; outcomes; accountability, transparency, and regulation; fairness and equity; and efficiency [13] (Table 1).

**Table 1:** WHO health system themes: data organization categories, subcategories, and indicators used.

System Evaluation Category	Subcategory	Description and Indicators
**Access and responsiveness**	Availability	Distance to facility and hours of service availability
	Timeliness of service	Waiting times from presentation to initial evaluation and subsequent testing, results, and follow-up
	Hospitality	Patient questionnaire responses regarding treatment of patients by the provider, and patient experiences when navigating the health system
**Quality**	Comprehensiveness of services	Availability of all components of WHO package of services
	Diagnostic accuracy	Rates of correct diagnosis on retrospective review
	Management standards	Rate of conformity to international disease-specific management standards
	Client retention	Rate of loss to follow-up or, alternatively, rate of appropriate patient return
**Outcomes**	Treatment success rates	Rate of therapy success, controlling for population characteristics and delayed presentation
	Population coverage	Proportion of catchment population reached by dedicated campaigns (e.g., vaccination rates)
	Morbidity	Rate of disability to patients, controlling for population characteristics
	Mortality	Rate of death among patients, controlling for population characteristics
**Accountability, transparency, and regulation**	Data accessibility and quality	Availability of data and appropriate use of indicators and statistics
	Public health functions	Contribution of healthcare system to core public health system functions (e.g., reporting of key diseases, preventative care)
	Reform capacity	Results of quality improvement initiatives
**Fairness and equity**	Financial barriers to care	User fees, under-the-table charges, and pharmaceutical costs
	Distributive justice	Healthcare availability commensurate with need
**Efficiency**	Cost	Absolute dollars spent for a given indication
	Redundancy	Repetition of diagnostic time, testing, supply chains, and therapy delivery
	Fragmentation	Separation of core healthcare system functions, generating sluggish management
	Delays	Time between ordering of tests or therapies and execution of tests and therapies

## Methods

### Search Strategy

We searched for primary literature in eight major databases using the indexed and free-text terms “private sector,” “privatization,” “public-private sector partnerships,” and “public sector” in various combinations, as described in Text S1. Because much of the discussion and data collection on this topic has been performed outside of academic circles by international agencies and non-governmental groups, we supplemented the database search by conducting the same keyword searches on the websites of the WHO library database WHOLIS, the World Bank Documents and Reports repository, the United Nations Children's Fund, the United Nations Development Program, the Bill & Melinda Gates Foundation, the Global Fund to Fight AIDS, Tuberculosis and Malaria, Oxfam International, and the Kaiser Family Foundation Global Health Division. The search terms included studies in English, French, Italian, Spanish, Portuguese, or Russian, published from 1 January 1980 through 31 August 2011.

### Study Selection

All titles and abstracts found by the search strategy were filtered for relevance to the study objective. Studies must have included data on a population in at least one low- or middle-income country, defined by the 2010 World Bank criteria of having current per-capita gross national income less than or equal to US$12,275 [14]. The full texts of potentially relevant articles were subject to the inclusion criteria listed in Table 2 to ensure they met basic minimum methodological standards. Qualitative studies were included if they specified a systematic methodology for interviews, focus group analysis, historical or political science analysis, or ethnographic observation (see Text S2 for the PRISMA checklist).

**Table 2:** Systematic review inclusion criteria.

Aspect	Minimum Criteria for Inclusion
Data collection in facilities	If comparison between public and private programs, comparators were randomly selected, or population matched/adjacent.
Sample size	For quantitative studies, must include >20 patients per facility or program described, or more than 100 persons if community-based household surveys. If questionnaire-based, must include >50% response rate.
	For qualitative studies, must include description of interviewees and systematic selection criteria.
Data description	For quantitative studies, must include data selection criteria, population demographic description, data collection method, and statistical analysis description.
	For qualitative studies, study must include population selection results based on specified criteria, data collection approach, and data synthesis strategy involving more than one author-reviewer if using a grounded-theory approach.
	For household surveys, study must include census of households or random selection from list of available households.
	For economics/cost-effectiveness studies, must specify data sources for costs and QALYs, specify model parameters and transition probabilities, conform to gold standards for CEA analysis [113] and specify discounting rates and method of summing costs across specified population.
Data presentation	Data and tables should add up and be consistent.
	Absolute numbers must be given, or denominators must be available for percentage results.
	Exclude if obvious data errors; inquire from authors in case of suspected typos.
	If statistical tests were performed, the tests need to be appropriate for the type of data being analyzed.
Bias	No other important issues in design, conduct, or analysis that could introduce bias considered on an individual basis, e.g., amount of potential bias if using different methods for collecting data between private and public providers.
	No unusual events occurred during study that could introduce bias.

CEA, cost-effectiveness analysis; QALYs, quality-adjusted life years.

### Data Extraction and Analysis

A data extraction method was designed by three reviewers (S. B., J. A., and D. S.). J. A. extracted the data using a preestablished standard data entry format into a database, with verification by S. B. to ensure consistency of coding. Standard data describing each study were also extracted, including the country where the study was performed, study period, study methodology, number of included participants, primary and secondary outcome measures and end points, and study limitations. Where disclosed, we noted the study funders and agencies. Disagreements between the two reviewers were resolved by consensus among all authors.

The data synthesis was structured into six themes from the updated WHO framework for health system assessment (see Table 1 for themes, subthemes, and indicators used to assess each theme) [13]. Relevant data that did not fall into one of these themes was separately included in the analysis in an “other factors” category that is discussed following the principal results. Reports containing information relevant to more than one theme were included in all related thematic areas. We did not perform further subanalysis of the highest quality studies as the authors could not agree to a vote-counting approach that would apply across the quantitative and qualitative methods and the six WHO themes captured in literature using different types of outcome variables.

## Results

**Figure 2:**
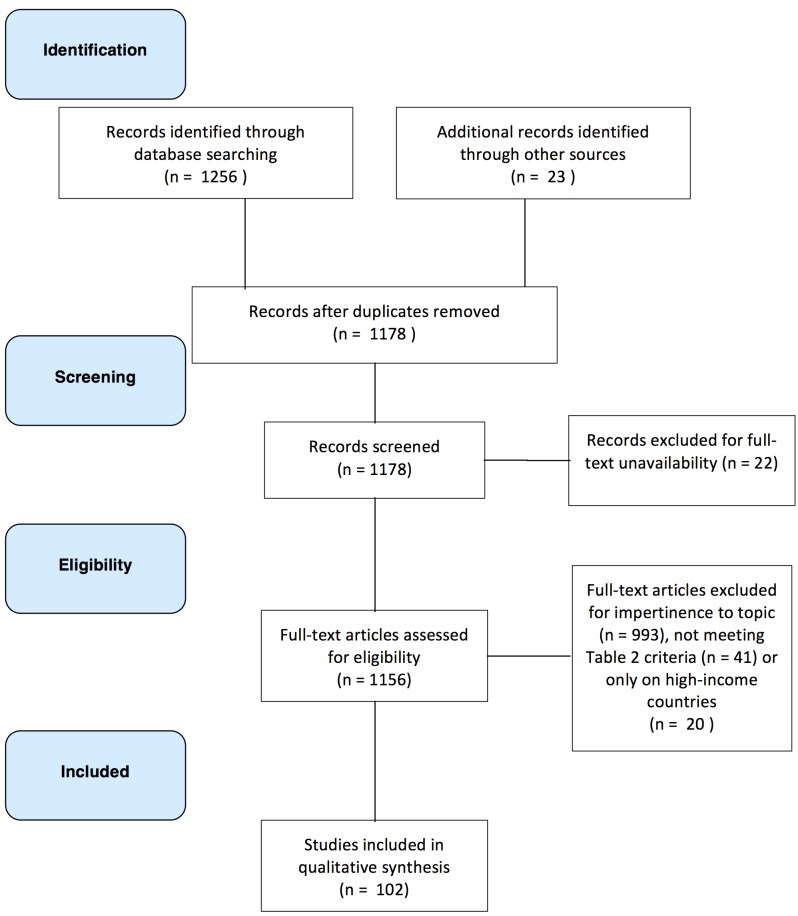
Flow diagram of study selection.

The study selection process is shown in Figure 2 as a PRISMA flow diagram. Of the 1,178 potentially relevant unique citations from all literature searches, 102 studies met the inclusion criteria. Key characteristics of the included studies are summarized in Table 3. Fifty-nine studies were empirical research studies and 13 involved meta-analysis, with the rest involving case reports or reviews. One-third of studies were carried out in the WHO-defined African region (*n* = 32) and another third in the Southeast Asian region (*n* = 34); most were published after 1990. We found that about nine out of ten studies directly compared quality of care in public versus private systems or assessed the demand for or utilization of services; the remaining studies examined drug availability or affordability or compared the cost and efficiency of services.

**Table 3:** Characteristics of included studies.

Characteristic	South Asia, East Asia, and Pacific	Sub-Saharan Africa	Latin America	Other	Multiple Continents/Not Context-Specific	Total
**Study year range**						
1980–1989	0	0	1	0	0	1
1990–1999	2	5	2	1	2	12
2000–2009	29	23	8	5	13	78
2010–2011	3	4	2	0	2	11
**Report type**						
Empirical research	29	21	5	4	0	59
Review/commentary	2	5	4	0	11	22
Meta-analysis/data synthesis	1	4	2	2	4	13
Case study	2	2	2	0	2	8
**Primary study purpose (research studies)**						
Describe or compare quality of private and public services	18	11	3	6	4	42
Assess drug availability and affordability	1	4	1	0	2	8
Assess demand for, access to, or utilization of services	13	14	9	0	11	47
Compare costs or efficiency of services	2	3	0	0	0	5
**Facility types**						
Hospitals	1	1	2	1	0	5
Outpatient clinics	3	4	0	1	0	8
Pharmacies	1	1	2	0	0	4
Multiple types	24	18	8	2	11	63
Not specified	5	8	1	2	6	22
**Service type**						
Promotive or preventive	3	3	1	1	1	9
Curative, rehabilitative, or palliative	20	15	5	3	4	47
All types	11	14	7	2	12	46
**Disease category**						
CD	19	17	4	2	4	46
NCD	3	2	5	1	2	13
Both CD and NCD	12	13	4	3	11	43
**Population age**						
Adults	7	12	6	2	2	29
Children	4	1	0	1	2	8
Both adults and children	23	19	7	3	13	65

CD, communicable disease; NCD, noncommunicable disease.

### Theme 1. Accessibility and Responsiveness

Six articles documented that a significant proportion of outpatient services in low- and middle-income countries appeared to be provided by the private sector [15]–[18]. However, the percentage of total visits varied substantially across countries and income levels [15]. In Viet Nam, the private sector provides 60% of all outpatient contacts. In India, more than 90% of children affected by diarrhea are taken to private healthcare providers, but the income gradient was not specified among studies reporting this data [17]. Among participants surveyed for HIV testing in 12 African countries, the proportion of patients using the private sector for testing ranged from 3% to 45% [19].

Several studies disaggregated utilization by income levels, tending to find that the private sector predominantly serves more affluent populations. A widely cited study on access of the private and public sectors was performed by the World Bank in 22 low- and middle-income countries using Demographic and Health Surveys [20]. Although interpretation of the findings varies [5],[20], the analysis found that in 19 of the countries studied, both wealthy and poor families received more care from the private than the public sector, but only when the private sector included private drug shops and similar informal providers [21]; when the composition of the private sector was limited to only licensed and certified healthcare personnel, the public sector provided the majority of care in low- and middle-income countries. However, there were three exceptions: Namibia, Tanzania, and Zambia, where private sectors are majority providers even when only licensed personnel are counted. The percentage of visits to the private sector was lower among the poor than among the wealthy in these surveys, but the difference was not statistically significant.

Additionally, in Colombo, Sri Lanka, where the private sector provided more than a quarter of all childhood immunizations overall, among the wealthiest quartile it provided 72% of immunizations but among the poorest quartile it provided only 3% [16]. In Uganda, 17.4% of women use private clinics or midwives for their family-planning-related medical care due to short distances and low transport costs, according to interviews conducted among 10,706 women, of whom 57% were in the country's lowest wealth quintile [18].

Few studies have investigated “accessibility” per se (i.e., the ability to access available services). However, wait times were consistently found to be shorter in private sector than in public sector facilities [22],[23]. One interview-based study in Ghana suggested that waiting times among public sector facilities could be longer for the same condition than private sector facilities by one or two hours [22]. Women living in rural Nigeria also reported preferring private obstetric services to public services because doctors were more frequently present at the time of patient presentation [23].

Patients tended to report worse hospitality from providers at public than private facilities (13 studies) [24]–[36]. In Bangladesh, for example, public providers ranked lower than private providers on scale-based surveys in which patients assessed the diagnostic explanation given them, courtesy of staff, cleanliness of facilities, capacity building, and the availability of certain medical inputs [36]. A study in India found that patients were seen for longer durations, were more likely to have a physical exam during their visit, and were more likely to have their diagnosis explained to them by private sector physicians than public sector ones [33]. Analysis in several countries suggested that patients in private sector facilities reported preferring the facilities because of shorter waiting periods, longer or more flexible opening hours, and better availability of staff [34].

### Theme 2. Quality of Health Care

Nine retrospective chart reviews and survey-based studies found that diagnostic accuracy and adherence to medical management standards were worse among private than public sector care providers [37]–[45]. Most of these studies examined infectious disease management protocols, including for tuberculosis and malaria [46]. Private practitioners had significantly worse knowledge of correct diagnosis and treatment. Other disease categories showed similar patterns of lower quality in the private sector. In Nigeria, public providers were significantly more likely to use rapid malaria diagnostics and to use the recommended combination therapies than private providers [47].

Similar poor adherence to guidelines in prescription practices, including subtherapeutic dosing, by private sector providers has been associated with a rise in drug-resistant malaria in Nigeria [47]. Parallel results were reported from Viet Nam [48]. In an analysis of outcome data from 24 countries, children with diarrhea were found to be less likely to receive appropriate oral rehydration salts and more likely to receive unnecessary antibiotics when seeing private providers than when seeing public providers [49]. However, a study of 119 private and ten public health clinics in Uganda found that both private and public providers prescribed antibiotics incorrectly (including not prescribing them when indicated), and in this study public providers were worse in adhering to national malaria treatment standards (14% versus 27%, *p* = 0.002) [45].

Poor adherence to guidelines in prescription practices, including prescribing subtherapeutic doses, failure to provide oral rehydration salts, and prescribing of unnecessary antibiotics were more likely to occur among private than public providers [47]–[49], although there were exceptions [45]. Higher rates of potentially unnecessary procedures, particularly cesarean sections (C-sections), were also reported at private than at public settings [50],[51]. One analysis of the Peruvian health system found significantly higher rates of C-sections after the privatization of delivery. The pre-reform rates in the private sector were already higher than the WHO recommended rate of 10%–15%; after reform, the rate exceeded 50%. The same has been found in South Africa, where 62% of women delivering in the private sector had C-sections, compared with 18% in the public sector [51]. Studies in Mexico suggested that fee-for-service payment structures (which are more heavily present in private than in public care delivery settings) incentivized increased C-sections [23].

Two cross-sectional studies documented a lack of drug availability and service provision at public facilities. A semi-structured questionnaire distributed to 24 health secretariats and directors of 39 city hospitals and 26 referral and teaching hospitals revealed that 76% of state facilities and 67% of city facilities lacked assisted reproductive technologies that were widely available in private sector facilities (though the exact percentage among such private facilities was not evaluated) [52]. In Tanzania, a semi-structured questionnaire distributed to 80 randomly selected patients and 45 health facility personnel staff working in diabetic clinics found that private facilities tend to stock more types of oral hypoglycemic agents than public facilities [53]. However, studies did not make clear whether the additional types of drugs were related to better outcomes or were simply additional brands of equivalent medication on hand.

Some studies of quality of care were performed in the private sector without having a comparative public sector group. Two studies in South Africa found that the majority of private general practitioners were not aware of the recommended medications, doses, or durations for treatment of sexually transmitted infections [54],[55]. Reviews in Nigeria and Laos reported similarly widespread use of ineffective therapies for malaria in the private sector [56],[57]. Sexually transmitted disease management in private clinics and drugs shops in Uganda revealed that 93% of cases were not properly managed per national guidelines, and the cure rate was 47% [58].

Dispensation of unnecessary medications and procedures was also reported to be higher among private sector providers according to four reports based on chart reviews. The most common incidents involved the unnecessary use of antibiotics for treatment of diarrheal diseases and non-complicated acute respiratory infections [32],[49]. Reports from Africa and Laos suggest ineffective and sometimes harmful pharmaceuticals are being distributed in the private sector [56],[57].

Surveys of patients' perceptions of care quality were mixed. While two survey-based studies suggested that patients perceived higher quality among private practitioners, possibly due to frequent prescribing of medications and more time spent with patients [20],[34], three interview-based studies suggested that patients perceived public sector healthcare workers as more competent [32],[59],[60].

### Theme 3. Patient Outcomes

Public sector provision was associated with higher rates of treatment success for tuberculosis and HIV [61]–[64] as well as vaccination [65],[66]. For example, in Pakistan, a matched cohort study in Karachi found that public sector tuberculosis care resulted in an 85% higher treatment success rate than private sector care [63]. In Thailand, patients seeking care in private institutions had significantly lower treatment success rates for tuberculosis, which was attributed to a three to five times greater likelihood of being prescribed non-WHO-recommended regimens than in the public sector [61]. In South Korea, tuberculosis treatment success rates were 51.8% in private clinics as opposed to 79.7% in public clinics, with only 26.2% of patients in private clinics receiving the recommended therapy, and over 40% receiving an inappropriately short duration of therapy [62]. Similarly higher rates of treatment failure were observed for private than public system patients on antiretroviral therapy for HIV in Botswana [64]. In India, an analysis of over 120,000 households, adjusted for demographic and socioeconomic factors, found that children receiving private health services were less likely to receive measles vaccinations [65]. Similar findings were reported from Cambodia [66].

Studies comparing pre- and post-privatization outcomes tended to find worse health system performance associated with rapid and extensive healthcare privatization initiatives. In Colombia, following major privatization reforms in 1993, population vaccine coverage declined for several diseases in the country, and tuberculosis incidence rose significantly [67]. In Brazil, privatization of fertility control services led to increased abortions, sterilization, and improper use of oral contraceptives (obtained without medical consultation), ultimately linked to higher mortality rates among young women [68]. However, a slower pace of privatization of health care services did not appear to correlate with a substantial worsening in patient outcomes among Latin American countries [69].

### Theme 4. Accountability, Transparency, and Regulation

Data on this theme tended to be unavailable from the private sector. No papers were found to describe any systematic collection of outcome data from entirely private sector sources. One recent independent review of Ghana's private sector referred to the private sector as a “black box,” with a dearth of information on delivery practices and outcomes [22]. Tuberculosis and malaria case notification to the public health system was particularly poor among private sector providers as compared to public providers in a number of countries [28],[48],[70]. However, while national vital statistics databases collected from public sector clinics and hospitals were widely available, they varied considerably in quality according to external assessments [22],[71].

Public–private partnerships also lacked data. A systematic review of data from public–private partnerships (including arrangements among governments and private, for-profit contractors) found few reported data that were of sufficient quality to assess the impact of partnership services and programs [72]. Poor data availability was observed in another systematic collection from several countries' private–public partnerships for sexual and reproductive health services. Most data available showed that after brief training of health providers, provider responses to questionnaires improved in accuracy, but no assessments were made of health outcomes [71]. An exception was a partnership in India that demonstrated increased birth attendant coverage from 27% to 53% over 7 mo among a cohort of 97,000 women [73].

Several reports observed significant public spending being used to regulate the private sector in order to improve patient care quality, particularly in African countries, and with limited effectiveness [22],[74]–[76]. The effectiveness of these regulations of the private sector was found to vary, often depending on public monitoring and enforcement [17],[34],[77]. Regulations to reduce the sale of unnecessary breast milk substitutes by private drug shops in Laos had limited impact until government inspectors visited sites to ensure appropriate sales and provided sanctions for legal violations [17]. In Indonesia, Kenya, Pakistan, and Bihar, clinical education programs to improve distribution of oral rehydration salts and reduce inappropriate antibiotic prescribing were found to have a greater impact when patients also received education, and when community healthcare workers were involved in monitoring, than when education was given only to clinicians [17]. Reviews in Zimbabwe and Tanzania identified anti-competitive practices and sales of inappropriate drugs [75]; attempted regulations in Zimbabwe were ineffective [76]. One review in Ghana indicated that the key public agency in charge of such regulation was unable to identify a large number of private providers in order to assess accreditation and quality: 2,612 of 11,430 drug shops were registered but had not received licenses [22]. A private–public partnership in South Africa to educate providers about national guidelines for sexually transmitted disease prevention and control had no effect on practice [77]. In Egypt a comparative assessment of clinical education programs found greater improvements in public sector practices than private sector practices [34].

### Theme 5. Fairness and Equity

Financial barriers to care, particularly user fees, were reported to be prevalent in both private and public systems. A World Bank study in Ghana concluded that there was no systematic evidence indicating whether user fees in the public sector were different than in the private sector [78]; however, the data presented showed that out-of-pocket user fees for patients were highest for private not-for-profit, lowest for public, and intermediate for private self-financed providers [22]. Hence, the conclusions of the report appear to be disputed by the data within the report.

As noted in the preceding sections, private sector health services tend to cater more greatly to groups with higher income and fewer medical needs (an illustration of the “inverse care law”), resulting in disparities in coverage [35],[79]–[85], although findings varied in several cases [86],[87]. Some studies suggested there was a systematic bias against indigent patients in terms of both quality and access. Exclusion of poor patients by the private sector was observed in South Africa [80] and Paraguay [81]. Poor patients were as likely as wealthier patients to seek care from private providers in Laos, but poorer patients received service from less qualified providers, with limited-quality services (no exam or advice, only medication dispensing) [35]. While most reports described income-based stratification in access, one report described stratification based on gender in addition to income. A nationally representative, cross-sectional, cluster-sample survey of 7,308 children in randomly selected rural and urban populations across Bangladesh observed that over 90% were taken to the private sector. However, when patients arrived at private clinics, children from higher income households and male children were significantly more often (*p*<0.001) directed to a licensed provider and treated with oral rehydration solution or an antibiotic than female or poor children [85].

Several studies suggested that the process of privatizing existing public services increased inequalities in the distribution of services. Analyses of the Tanzanian and Chilean health systems found that privatization led to many clinics being built in areas with less need, whereas prior to privatization government clinics had opened in underserved areas and made greater improvements in expanding population coverage of health services [82]–[84]. Privatization in China was statistically related to a rise in out-of-pocket expenditures, such that by 2001, half of Chinese surveyed reported that they had forgone health care in the previous year due to costs; out-of-pocket expenses accounted for 58% of healthcare spending in 2002 compared with 20% in 1978 when privatization began. The cost burdens of privatization related to an increase in disparities in healthcare coverage and infant mortality between urban and rural areas [79]. One survey-based study using Demographic Health Survey data from 34 sub-Saharan African countries found that privatization was associated with increased access, and reduced disparities in access between rich and poor [86]. A second analysis of the same dataset, however, found no change in inequality in use of modern contraceptives with the expansion of the private sector [87].

Private contracting and social franchises showed potential for expanding private sector coverage to impoverished groups, although conclusions are tentative because comparisons to the public sector were unavailable. One World Bank study in Cambodia reported improvements in healthcare coverage in poor districts after contracting out services to private companies specifically to increase coverage. When contracts explicitly included targets for reaching the poor, contractors improved health services for the most marginalized groups, although comparison was not made to the results of a similar investment in public sector services [88]. Several related World Bank initiatives took the form of social franchises, in which private providers pay a fee and are provided training, managerial assistance, and certification in a provider network [20],[89],[90]. Several case studies of social franchises [20],[89],[90] found higher care utilization among the lower socioeconomic groups of private franchisers than of control private clinics for contraceptive use, HIV counseling, antenatal care, and vaccination [17],[91],[92].

### Theme 6. Efficiency

Several reports observed higher prescription drug costs in the private sector for equivalent clinical diagnoses [33],[36],[53],[67],[93]–[96]. In a survey study of prescription costs in India, costs were higher for every class of visit in the private sector [33]. Two-thirds of outpatients in the private sector, compared with one-third in the public sector, received an injection for similar presentations, but the study did not investigate what fraction was unnecessary [33].

Both generic and brand-name drugs were found to be higher in price in the private sector [96]. Tanzanian private facilities typically used more brand-name oral hypoglycemic agents, but even generic medications were five times higher in price [53]. Similar findings were reported in India [96]. A study in Bangladesh found that private sector healthcare prices in the country—not just those associated with medications—have been growing far above the inflation rate [36].

There is also evidence that the process of privatization is associated with increased drug costs [36],[53],[67],[93],[94],[96]. A study of the Malaysian health system found that increasing privatization of health services was associated with increased medicine prices and decreased stability of prices [93]. Healthcare costs in Colombia rose significantly following privatization reform in 1993, and 52% of capitation fees were spent on administration [67]. Similar privatization in some parts of South Africa were associated with a 13% to 32% cost increase in overall health spending, without associated increases in coverage or indications [94]; costs of prescriptions were significantly lower in the public sector, likely due to generic substitution, prepackaging of medications, and use of treatment protocols [95].

Higher drug costs are in part associated with disease complications attributable to delayed diagnosis or incorrect disease management [97],[98]. In Bolivia, seeking care in the private sector was associated with longer delays in tuberculosis diagnosis and greater costs [97],[98]. It was estimated that in Mexico, Brazil, and South Africa, unnecessary C-sections increased delivery-related health costs in the private sector by at least 10-fold [23]. In Bangladesh, private contracting of health services appeared to increase costs related to complications and delays in service access [36].

Several World Bank studies found significant fragmentation in purchasing and distribution across and within the public and private sectors, resulting in higher drug prices and redundant treatments that increase overall healthcare costs [22],[99]. The absence of reliable distributors for pharmaceuticals in a study in Ghana led to several intermediary groups being used to distribute medications, increasing prices between 5% and 200% [22]. The large number of small-scale hospitals and clinics in some sub-Saharan African countries fragmented delivery, such that patient diagnoses and treatment histories were unavailable between institutions [22],[99], often significantly delaying care, and resulting in redundant tests and sometimes administration of incorrect medication to patients. Several private primary care providers reported difficulties referring their patients to public sector secondary care facilities, as public facilities did not accept the diagnoses made by the private providers and often required the patient to restart the consultation process [99].

Competition between public and private delivery tended to decrease drug prices. One large multilevel analysis of the content and cost of 700 medication transactions observed in 14 private and public settings in Mali revealed that private providers were more likely to prescribe brand-name drugs, injectable drugs, and more antibiotics; however, the availability of drugs in the public sector decreased prices in the private sector [100].

Contracting of public healthcare services to private providers has also been estimated by the World Bank to reduce costs of and waiting times for contracted services [36],[101], although the effects of contracting differ markedly by the type of healthcare service and across countries [17],[102]. In Cambodia, contracted districts had costs of $22.7 per person per year versus $26.4 among non-contracted districts, although there were no tests of statistical significance [36]. One highly cited secondary analysis reported this outcome as a 17% savings resulting from contracting [101]. Peer-reviewed studies of contracting in Zimbabwe and South Africa found that costs were unchanged by contracting in South Africa but were lower after contracting in Zimbabwe [17]. One review of contracting experience in Madagascar and Senegal found that large expenditure from public sector ministries was necessary to manage and supervise private contracts, increasing overall costs in those two countries by 13% and 17%, respectively [102].

### Other Observed Factors

A few key findings reported in articles did not clearly fit into the WHO health system themes, mainly involving recent reports of complex “competitive dynamics” between private and public health sectors. First, a “crowding out” effect appeared to occur between private and public sector services for expanding delivery. This process involved the transfer of public funds and personnel to private sector development, followed by reductions in public sector service budgets and staff availability. In Ghana, new private services in urban middle- and upper-socioeconomic populations were found to reduce revenues for public sector hospitals that also provided care to poorer populations [22]. At times, however, the process was a passive privatization: public sector funds were increasingly allocated to private–public partnerships without accompanying shifts in demand, so that the public sector's effective budget per patient was reduced. This dynamic was observed in post-apartheid South Africa [103], as well as in Uganda [104] and Brazil [105]. Public–private partnerships and private contractors were often involved in such scenarios, but did not typically disclose the data necessary to fully evaluate these arrangements.

Public and private sector interactions also had implications for delivery, staffing, and disease control. Interviews of Indian patients suggested that several private practitioners who work in both public and private sectors advised patients to visit their private clinics or requested further payments in order to continue providing care in the public clinic [106]. Doctors tended to migrate towards private sector and urban jobs, depriving the public sector and rural areas of physicians [107]. However, private hospital systems often subsidize or provide healthcare technologies to patients who cannot obtain these services from public hospitals. For example, in Botswana, private hospitals often receive cancer patients from public hospitals that are unable to provide radiation oncology services [78]. In some cases, however, the services in differing sectors undermined performance of one or both sectors. Several studies found that poor reporting of diseases in the private sector impeded public sector control of communicable diseases [28],[48],[70].

## Discussion

Our systematic review of comparative analyses of public and private healthcare systems in low- and middle-income countries found strengths and limitations in both sectors for each of six main WHO health systems framework themes. Private sector healthcare systems tended to lack published data by which to evaluate their performance, had greater risks of low-quality care, and served higher socio-economic groups, whereas the public sector tended to be less responsive to patients and lacked availability of supplies. Contrary to prevailing assumptions, the private sector appeared to have lower efficiency than the public sector, resulting from higher drug costs, perverse incentives for unnecessary testing and treatment, greater risks of complications, and weak regulation. Both public and private sector systems had poor accountability and transparency. Within all WHO health system themes, study findings varied considerably across countries and by the methods employed.

The review has several limitations, which reflect the existing data and literature purporting to compare the healthcare performance of public and private sectors. First, existing studies have focused on isolated topics where data are more abundant, and as a result have overlooked important dimensions of health sector performance. To address this limitation, we drew on a broader range of data, including reports from non-governmental organizations and international agencies like the World Bank. This step was particularly important for acquiring data from the private sector, since such data are relatively unavailable in the peer-reviewed academic literature. Thus, some studies included were not peer-reviewed. Our review involved a detailed analysis of methodological criteria for these studies to ensure they met similar standards of data analysis and reporting as peer-reviewed research. Second, although it was not possible to perform a quantitative meta-analysis because of variations in coding and outcomes, we were able to identify unsubstantiated claims in several cases, which appeared more prominent among non-peer reviewed sources. For example, the World Bank has made strong claims that investing in public–private partnerships will improve efficiency and effectiveness in the health sector [108], yet several of its publications revealed that these assertions were either unsupported by data or the data was not provided in sufficient detail to pass minimal inclusion criteria required for this review [20],[78]. Efforts are needed to address potential conflicts of interest of such agencies and their implications for research and data reporting, particularly as their analyses are often very highly cited in the academic literature on health system assessment and performance.

Third, our reliance of the WHO health system themes enabled the analysis to address systematically and comprehensively the existing research on public and private sectors. However, a limitation of the thematic framework, for example, is that several elements of the patient experience in healthcare settings, such as waiting times, are not systematically cataloged in current assessments. This implies that future research in the area should include a focus on how experiential aspects of care are relevant to healthcare seeking and outcomes (such as the likelihood of follow-up among patients requiring return visits) for differently structured care environments. Fourth, the review identified mixed results in several cases and was unable to account for a range of potential modifying factors, partly as a limitation of the broad WHO health system components that do not incorporate contextual factors. For example, treatment of infectious diseases in public settings may be more efficient than in private settings because of higher volume, and greater use of systematized protocols due to that higher volume. Such differences limit the ability of existing work to compare fairly the public and private sector for differing disease categories and in differing social and economic contexts of healthcare delivery.

Although it was not the focus of our research, we observed that some of our findings in low- and middle-income countries mirrored existing evidence from high-income countries. For example, the lack of data from private sector groups was similar to the situation in the UK, where the privately run Independent Sector Treatment Centres was unable to provide healthcare performance data when required [109]. However, our evidence also indicates that contextual factors modify the relationships we have observed, so that it is not straightforward to transpose health system evidence from high-income countries to low- and middle-income countries. Importantly, we observed that regulatory conditions interact with the effectiveness of public and private sector provision, but in low- and middle-income countries regulatory capacity is much weaker. As one example, the reviewed data suggest that systems that incentivize more procedures (rather than better outcomes) tend to lead to inefficiencies and poorer health outcomes. One extensively studied alternative system in high-income countries is pay-for-performance remuneration systems. It remains unclear what effects such programs may have in low- and middle-income countries as compared to high-income countries.

Our study has important implications for future research and policy. Future research is needed to address several important methodological limitations of existing studies. Many analyses were excluded from the review because they lacked a systematic approach to cataloging health system quality. Ideally, analyses should be comparative and should include a “counterfactual” in order to make causal claims about the effects of the particular benefits of providing services in one sector or the other. For example, social franchising to engage private providers in an organized regulatory system, which has been extensively piloted, has yet to be analyzed over the long term using outcome data and a comparison with commensurate investment in public sector development [88]. Studies also need to specify carefully the definition of the private and public sectors. When the private sector included unlicensed physicians, it was found to provide the majority of coverage for low-income groups, but when only licensed providers were included, the public sector was found to be the main source of healthcare provision in low- and middle-income countries. While some commentators report a higher number of absolute healthcare workers in the private sector, and a higher number of visits among the population to the private sector, these observation may be artifacts of improperly coding a large portion of private “providers” who are not actually qualified healthcare personnel, but rather drug store salespeople [1],[5]. Most studies fail to capture the full scope of effects of reforms on the healthcare system, focusing on an isolated health system component. A reform may enhance public sector performance but compromise the market in the private sector, or vice versa. Standards may need to be developed for health system research for identifying what is “safe” and “effective” overall for patients across socioeconomic strata, just as we do for pharmaceutical safety and efficacy.

Some authors have highlighted the lack of regulatory infrastructure available in low- and middle-income countries to monitor the performance of private healthcare contractors [110]. Despite the lack of data about private sector performance, recent initiatives by the World Bank's International Finance Committee are underwriting the expansion of private sector services among low- and middle-income countries. For example, in sub-Saharan Africa, the International Finance Committee has created a private equity fund to make 30 long-term investments in private health companies. These conflicts of interest pose a potential threat to the validity of World Bank–sponsored studies and raise the need for independent scrutiny.

Our review indicates that current data do not support claims that the private sector has been more efficient, accountable, or medically effective than the public sector [8]. The review also identifies several areas of focus for quality improvement. In the private sector, benefits may accrue from enhancing medical knowledge for appropriate diagnosis and disease management, drawing on specific quality improvement programs for continuing medical education that may serve as models [17]. It is also important to address conflicts of interest from physician-induced demand, particularly when prescribers are also drug store owners. Regulation and consumer education have been more successful than a reliance on clinical education alone in Pakistan and Bihar [17]. In the public sector, quality improvement may need to address incentives to perform at high standards among providers who may not feel threatened by a lack of business in the manner that private practitioners do. One proposed approach is to link provider compensation with results from patient outcomes, weighted by baseline disease risk in the patient population [111]. More generally, policy research needs to determine how targeted interventions might address these core weaknesses among both private and public delivery environments, including the lack of disclosure of outcome and performance data; as a measure of accountability, public transparency can be considered a vital sign of system performance (particularly for those systems receiving public subsidies; [112]). While there is no clear definition of a “basic minimum dataset” for countries to capture health sector performance, we did notice several common themes in our data review. In many of the countries studied, surveillance of disease treatment outcomes among adults, and particularly noncommunicable disease, was found to be limited. Furthermore, we found further data gaps in health system performance around the issues of waiting times, financing changes (e.g., to further characterize the “competitive dynamics” we described), and outcomes of quality improvement efforts within each sector.

A critical challenge in years to come is how to address competitive dynamics between private and public realms, so that public sector facilities are not stripped of resources that are given to the private sector as subsidies, and so that the ability of public clinics and hospitals to retain skilled healthcare workers is not compromised, especially as both types of systems attempt to coexist in the healthcare delivery environment of low- and middle-income countries. These findings are consistent with earlier findings of an “infrastructure inequality trap” in some countries [103], in which government funding is increasingly attracted towards private hospitals and away from the public sector hospitals. This occurs when private patients can afford to pay for greater infrastructure at private hospitals. Those hospitals then report greater “absorptive capacity” for future funds, and higher numbers of healthcare personnel, thereby attracting more funding from government institutions, shifting budgets away from public sector facilities that struggle to maintain human and physical infrastructure. Furthermore, we found evidence that many public–private initiatives involve public sector funding being dedicated to monitoring and preventing corruption in the private sector.

Overall, the data describing the performance of public and private systems remains highly limited and poor in quality, suggesting that further investigations should more systematically make data available to track the performance of both public and private care systems before further judgments are made concerning their relative merits and risks.

## Supporting Information

Text S1 **Search strategy.**
(DOC)

Text S2 **PRISMA checklist.**
(DOC)

## Abbreviations

C-section: cesarean section
WHO: World Health Organization
