# Supplementary material for: Comparative Performance of Private and Public Healthcare Systems in Low- and Middle-Income Countries: A Systematic Review
Source: PLoS Med. 2012 Jun 19;9(6):e1001244. doi: 10.1371/journal.pmed.1001244 (PMC3378609; doi:10.1371/journal.pmed.1001244)
Supplement: Text S1 — Search strategy. (DOC) [file pmed.1001244.s001.doc]

**Supporting Information 1: Search strategy**

We searched the following electronic databases:

- Medline
- EMBASE
- Web of Knowledge
- African Index Medicus
- Eastern Mediterranean Literature-WHO
- IndMED
- Index Medicus for South-East Asia Region
- LILACS

We used the following combination of terms:

Search #1: "private sector"[MeSH Terms] OR privatization[Text Word] OR “public-private sector partnerships”[MeSH Terms];

Search #2: "public sector"[MeSH Terms]

Search #3: #1 AND #2 AND (Portuguese[lang] OR Russian[lang] OR English[lang] OR French[lang] OR Spanish[lang] OR Italian[lang])

Search #4: #1 AND #2 AND #3 AND NOT (Letter[ptyp] OR Editorial[ptyp]);

The search was performed in September 2011 and included articles from January 1980 through August 2011. Of note, none of the articles fulfilling this criteria were in non-English languages.
